# Secondary punctate inner choroidopathy in an RP2 associated retinopathy female carrier

**DOI:** 10.1186/s12348-026-00590-1

**Published:** 2026-05-04

**Authors:** Gonzalo Roig-Ferreruela, Sofía Uncetabarrenechea-Larrucea, María Aramberri-Araiz, Sonia Valsero-Franco, Joseba Artaraz, Ester Carreño, Alex Fonollosa

**Affiliations:** 1 Department of Ophthalmology, Fundación Oftalmológica Médica de la Comunidad Valenciana (FOM), Avenida Pío Baroja 12, Valencia, 46015 Spain; 2 Department of Ophthalmology, Biocruces Bizkaia Health Research Institute, Cruces University Hospital, University of the Basque Country, Barakaldo, Spain; 3 Hospital Universitario La Paz, Madrid, Spain; 4 Department of Retina, Instituto Oftalmológico Bilbao, Bilbao, Spain

**Keywords:** Punctate inner choroidopathy, Secondary punctate inner choroidopathy, Retinitis pigmentosa, X-linked retinitis pigmentosa, Retinal dystrophy

## Abstract

**Purpose:**

To describe a case of punctate inner choroidopathy (PIC) occurring in a RP2 associated Retinopathy female carrier, highlighting the diagnostic contribution of multimodal imaging and the therapeutic course.

**Methods:**

Observational case report with multimodal retinal imaging, electrophysiology, and genetic testing.

**Results:**

A 46-year-old woman with high myopia (− 8 diopters in both eyes) and a right Adie’s pupil presented with a shadow-like visual disturbance in the right eye (RE). Family history was notable for X-linked retinitis pigmentosa: her brother was hemizygous for the pathogenic RP2 variant c.1 A > G (p.Met1?), and her mother was a heterozygous carrier with light perception vision in both eyes. At presentation, best-corrected visual acuity (BCVA) was 20/32 in the RE and 20/20 in the left eye (LE). Fundus examination revealed diffuse chorioretinal atrophy and multiple punched-out lesions, predominantly nasal, with some macular involvement suggestive of inactive PIC. Subtle peripheral bone spicules were also observed. Fundus autofluorescence (FAF) demonstrated hypoautofluorescent lesions corresponding to the punched-out scars and radial hyperautofluorescent streaks around the posterior pole, a characteristic pattern of X-linked RP carriers. Flash electroretinography revealed bilateral rod and cone dysfunction, more pronounced in the LE. Genetic testing confirmed the heterozygous RP2 c.1 A > G variant, establishing the patient as an X-linked RP carrier. Two years later, the patient developed a new greyish inflammatory lesion in the papillomacular bundle of the RE. OCT demonstrated hyperreflective subretinal material disrupting the RPE with posterior hypertransmission, consistent with an acute PIC lesion. She was treated with systemic corticosteroids and methotrexate. Subsequent flares required treatment escalation, including mycophenolate mofetil and later a combination of methotrexate and adalimumab due to intolerance. One year after initiating biologic therapy, the patient remains in remission with stable visual acuity (20/32 RE, 20/25 LE).

**Conclusions:**

Secondary PIC may occur in carriers of X-linked RP. Multimodal imaging and genetic testing are essential for accurate characterization, and immunosuppressive therapy can achieve sustained disease control.

## Introduction

Punctate inner choroidopathy (PIC) is an uncommon, idiopathic, presumably autoimmune disorder of the chorioretina that primarily affects young myopic women. Recently, the so-called secondary PIC has been described [1]. In this entity, typical inflammatory PIC lesions develop in patients with underlying chorioretinal diseases including hereditary retinal conditions. Regarding the pathogenesis of secondary PIC, it is hypothesized that a damaged outer retinal barrier could expose new retinal antigens to the immune system, triggering a local inflammatory process [2]. Herein, we describe a patient carrier of a RP2 gene variant, with mild structural retinal abnormalities, who exhibited typical PIC inflammatory lesions. Our observation, to our knowledge not previously described in the literature, expands the spectrum of retinal diseases associated with secondary PIC, and suggests that even a mild damage of the outer retina may serve as a trigger for local autoimmune reactions.

## Case report

**Fig. 1:**
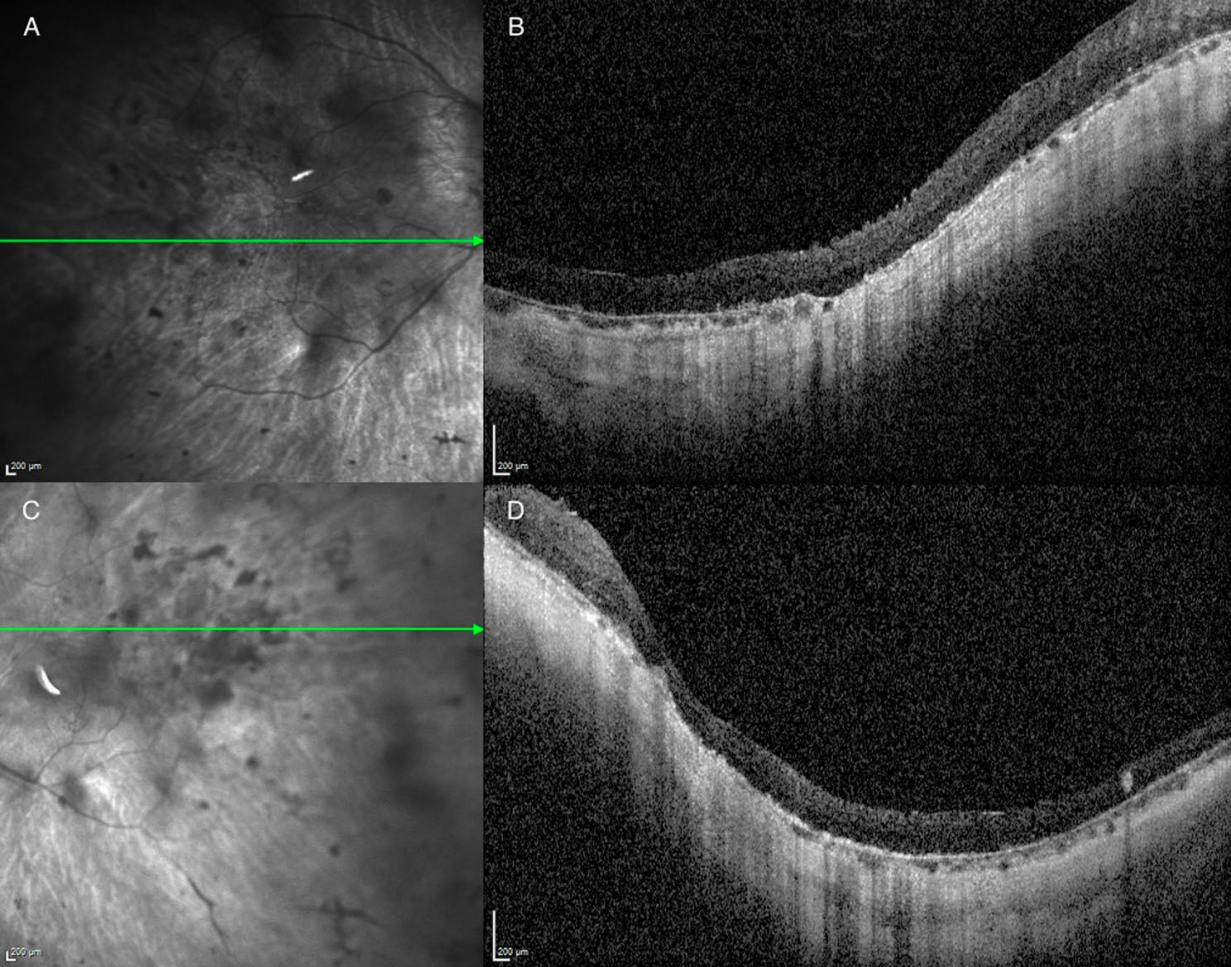
**A)** Infra-red Retinography of brother’s patient right eye (RE). **B)** OCT of brother’s patient RE. **C)** Infra-red Retinography of brother’s patient left eye (LE). **D)** OCT of brother’s patient LE

**Fig. 2:**
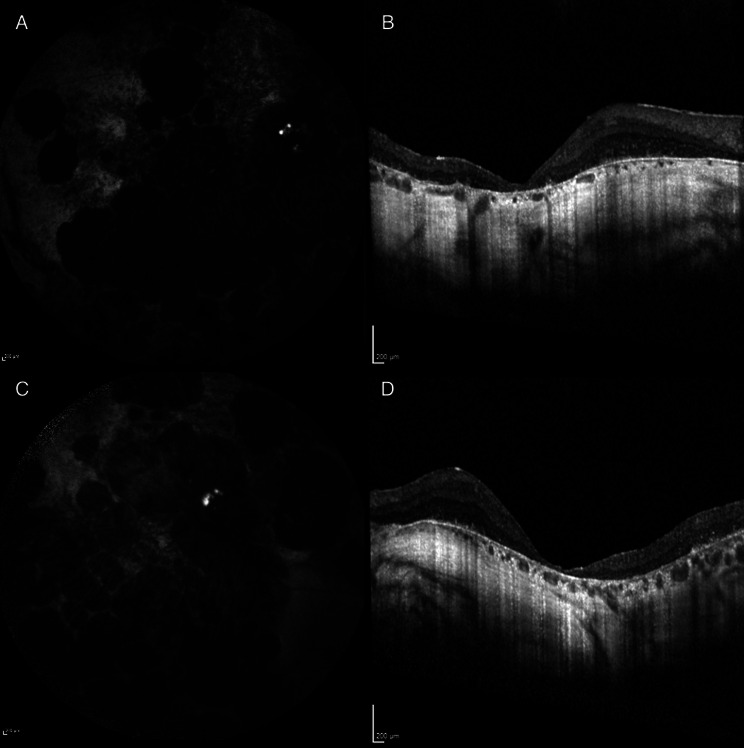
**(A)** FAF of mother’s patient right eye (RE). **(B)** OCT of mother’s patient RE. **(C)** FAF of mother’s patient left eye (LE). **(D)** OCT of mother’s patient LE

A 46-year-old woman with a history of high myopia (− 8 diopters) in both eyes and a right *Adie’s pupil* was referred to our hospital with a shadow-like visual disturbance in her right eye (RE). Regarding familiar history, her brother suffered from X-linked RP. He was hemizygous for the *RP2* gene variant c.1 A > G (p.Met1?), classified as pathogenic and associated with X-linked RP. Figure 1 shows brother’s Optical coherence tomography (OCT) and Infrared Retinography. Moreover, her mother was heterozygous for the same variant and had light perception visual acuity in both eyes. Figure 2 shows mother’s OCT and fundus autofluorescence (FAF) findings. These results had confirmed an *RP2*-related (*300757) RP, and also consanguinity in the family.

**Fig. 3:**
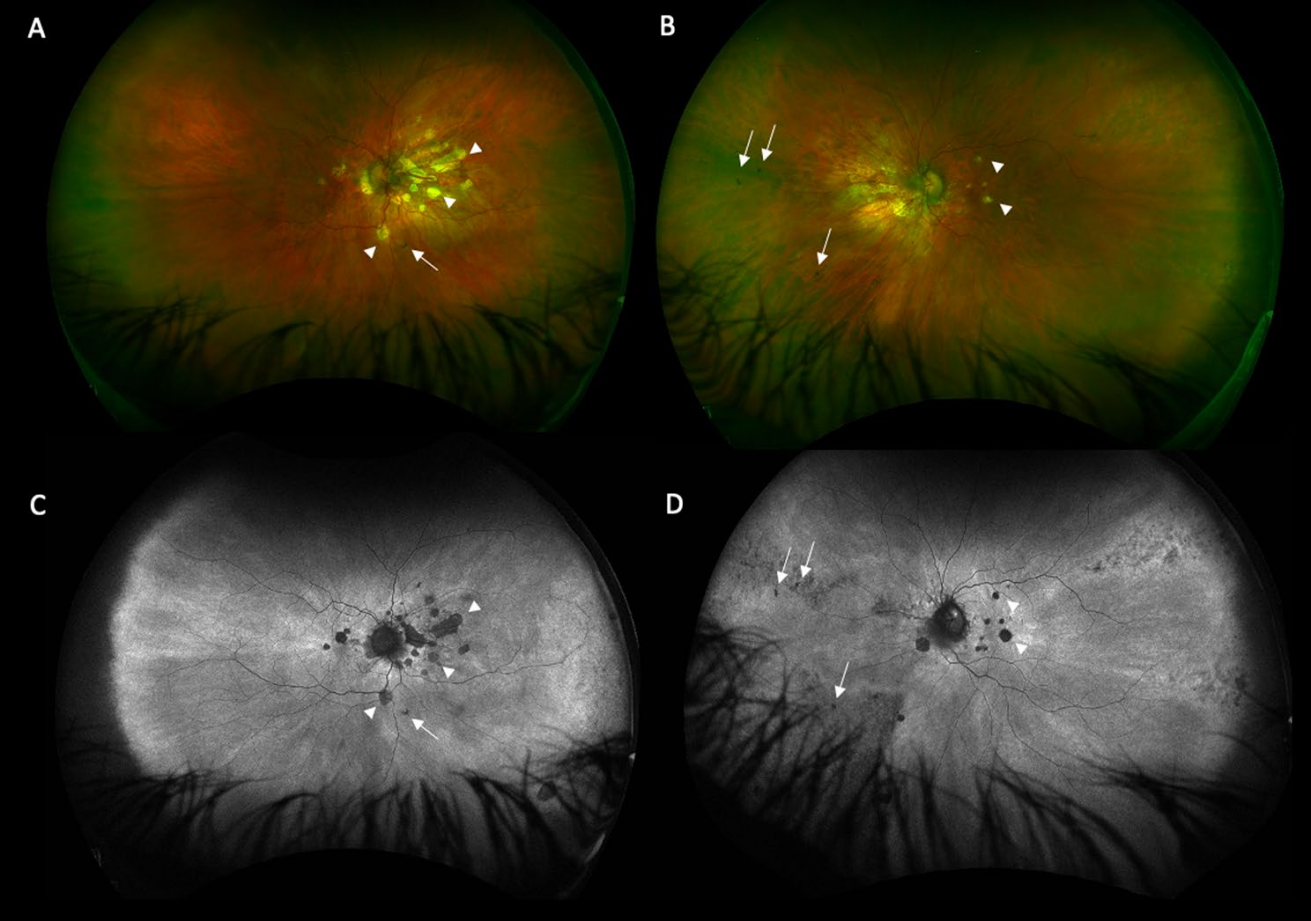
**(A)** Baseline fundus of the right eye. White arrows: bone spicules. Arrow-heads: PIC lesions. **(B)** Baseline fundus of the left eye. White arrows: bone spicules. Arrow-heads: PIC lesions. **(C)** Baseline fundus autofluorescence of the right eye. White arrows: bone spicules. Note radial hyperautofluorescent lesions around the posterior pole. Arrow-heads: PIC lesions. **(D)** Baseline fundus autofluorescence of the left eye. White arrows: bone spicules. Note radial hyperautofluorescent lesions around the posterior pole. Arrow-heads: PIC lesions

**Fig. 4:**
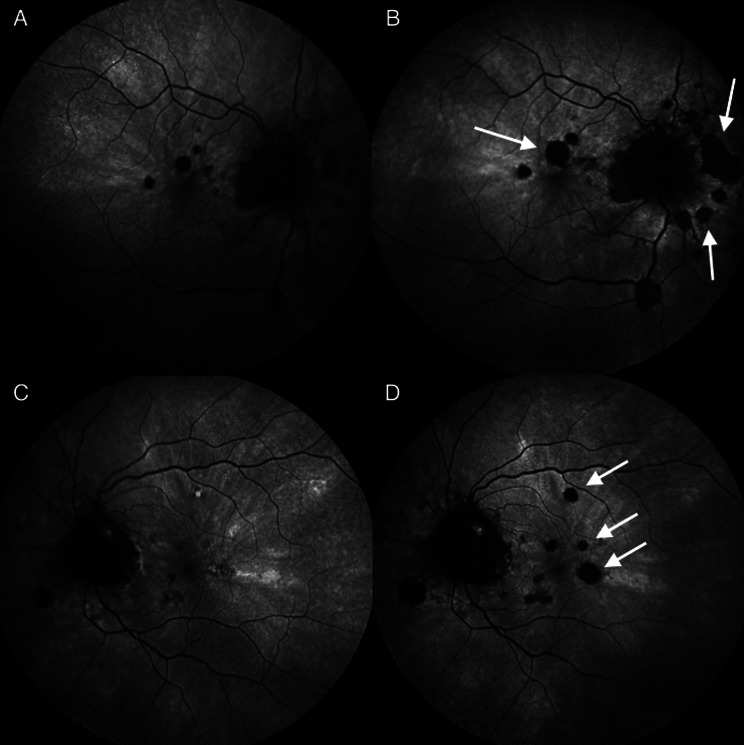
**(A)** Baseline fundus autofluorescence of the right eye. **(B)** Fundus autofluorescence of the right eye two years later. White arrows indicate the growth of characteristic PIC lesions. **(C)** Baseline fundus autofluorescence of the left eye. **(D)** Fundus autofluorescence of the left eye two years later. White arrows indicate the appearance of characteristic PIC lesions

**Fig. 5:**
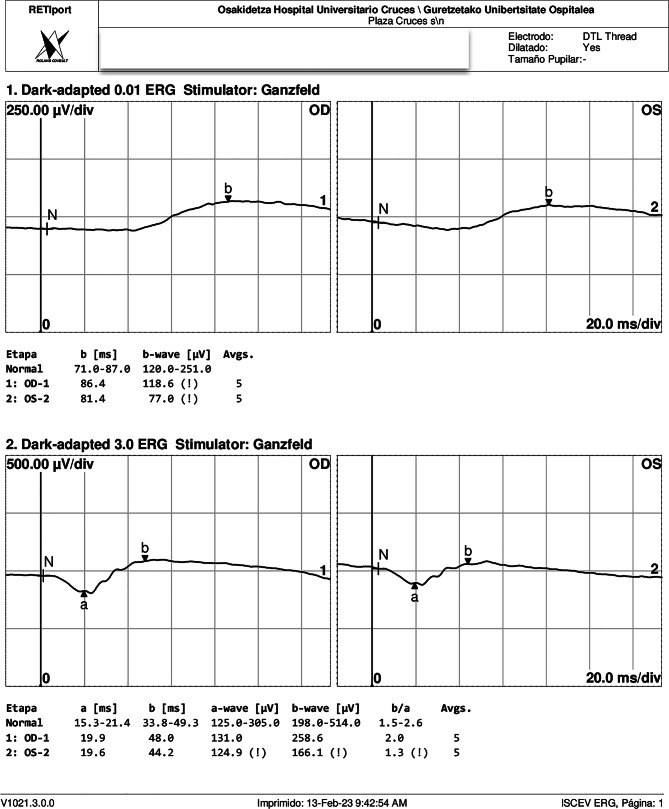
Full-field electroretinography (ffERG) was performed according to ISCEV standards. Scotopic responses demonstrated mildly reduced rod-specific b-wave amplitudes in both eyes, more pronounced in the left eye. The dark-adapted 3.0 and 10.0 responses showed relatively preserved a-wave amplitudes in the right eye and borderline reduction in the left eye, with mild reduction of b-wave amplitudes in the left eye but no electronegative configuration. Oscillatory potentials were within normal limits bilaterally. Photopic responses revealed reduced cone a-wave amplitudes in both eyes, while b-wave amplitudes and 30-Hz flicker responses were within normal limits with preserved timing. Overall, the ERG findings are consistent with mild, bilateral photoreceptor dysfunction, slightly asymmetric (OS > OD), predominantly affecting rod function with subtle cone involvement. These findings are compatible with a carrier state of X-linked retinitis pigmentosa rather than a manifest, generalized rod–cone dystrophy

**Fig. 6:**
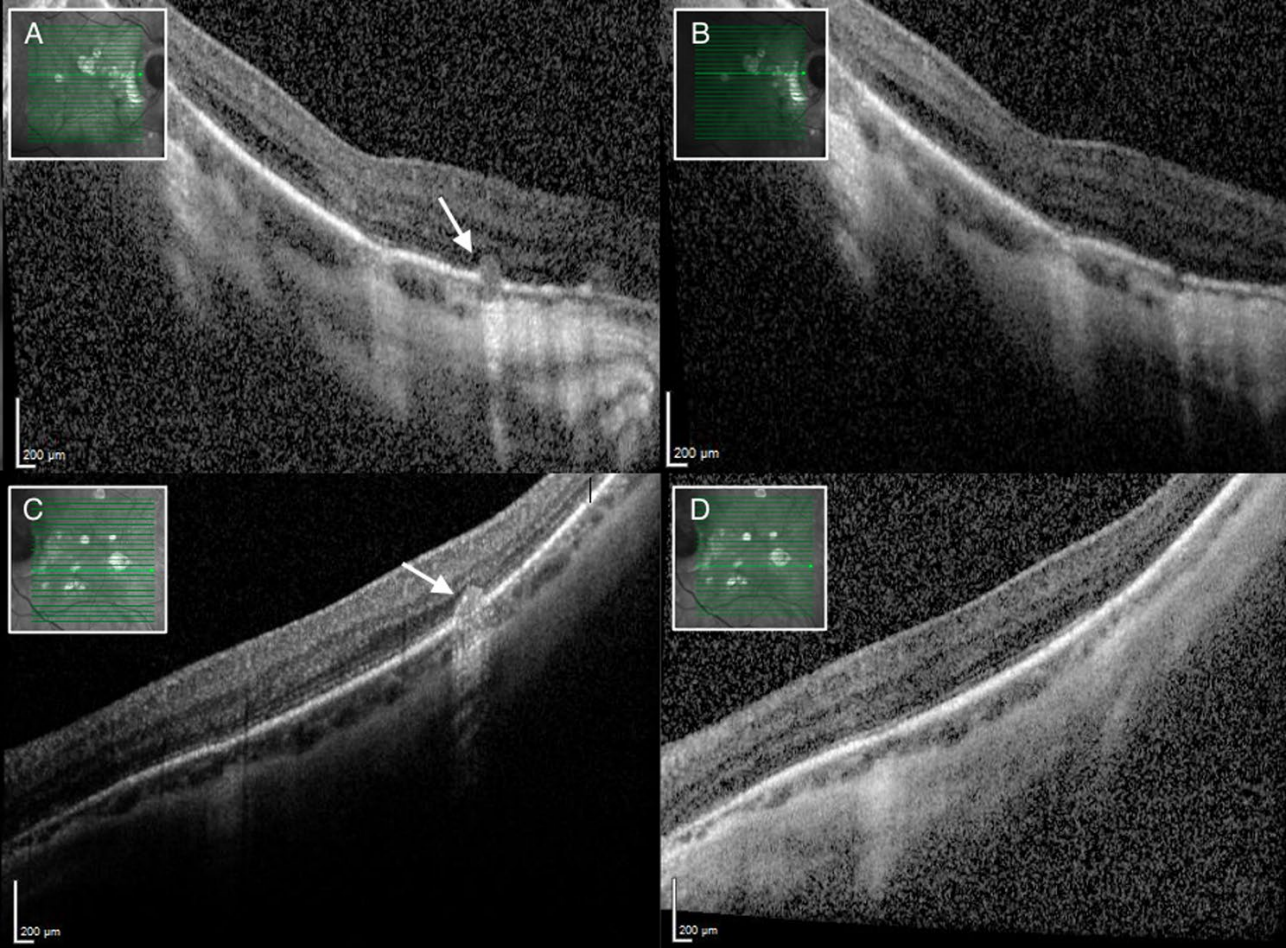
**(A)** OCT of the right eye with choroiditis flare. White arrows indicate the choroiditis flare focus. **(B)** OCT of the right eye one month after with choroiditis flare solved. **(C)** OCT of the left eye with choroiditis flare. White arrows indicate the choroiditis flare focus. **(D)** OCT of the left eye one month after with choroiditis flare solved

On evaluation, Best-corrected visual acuity (BCVA) was 20/32 in RE and 20/20 in LE. No relevant findings were observed in the anterior chamber or the vitreous. Intraocular pressure (IOP) was 15 mmHg in both eyes. Fundoscopy revealed diffuse chorioretinal atrophy and punched-out lesions predominantly in the nasal area of both eyes, and a few in the macula, suggestive of inactive lesions of PIC. A few subtle bone spicules were also observed in the periphery (Fig. 3). OCT showed choroid and outer retinal thinning without signs of choroidal neovascularization (CNV). FAF imaging demonstrated hypoautofluorescent foci corresponding to punched-out lesions and, more remarkably, radial hyperautofluorescent lesions around the posterior pole, typical of X-linked RP carriers. (Fig. 4). Accordingly, flash electroretinogram (fERG) revealed mild, bilateral photoreceptor dysfunction, slightly asymmetric (OS > OD), predominantly affecting rod function with subtle cone involvement (Fig. 5) Genetic analysis was seeked, which disclosed the presence of the heterozygous variant c.1 A > G, p.(Met1?) in the RP2 gene (chromosome X), and thus confirming the X-linked RP carrier status of the patient.

After 2 years without symptoms, the patient consulted again for a new shadow-like visual disturbance in the RE. Fundus imaging revealed previous atrophic areas in the macular and nasal region, and a new greyish inflammatory lesion in the papillomacular bundle, consistent with an acute PIC flare. OCT scans over this area demonstrated hyperreflective material breaking through the RPE, with posterior hypertransmission, typical of an acute PIC lesion. (Fig. 6) She was treated with systemic corticosteroids (3 pulses of 250 mg methylprednisolone followed by oral prednisone taper, starting with 20 mg/day) and initiated on subcutaneous methotrexate (15 mg/week).

During one of the routine follow-up examinations, four months after the initial episode, OCT revealed acute lesions at the margins of pre-existing atrophic areas, this time in the LE (Fig. 6). We treated this acute flare with the intensification of oral steroids. Therefore, we decided to switch the immunosuppresive regimen to mycophenolate mofetil 2 g/day.

Four months after she had several febrile episodes that were thought to be related to intolerance to this therapy so treatment was changed back to methotrexate (10 mg/week) and adalimumab (40 mg/2 weeks) was added.

One year after initiation of immunosuppressive treatment the patient remains in remission under adalimumab and low-dose prednisone (5 mg/day) and methotrexate (10 mg/week) with BCVA 20/32 in RE and 20/25 in LE.

## Discussion

This case illustrates a compelling example of secondary PIC arising in the context of an underlying inherited retinal disorder. The patient, a heterozygous carrier of an *RP2* pathogenic variant, exhibited structural and functional features consistent with RP2 associated retinopathy. Female carriers of RP2 variants are known to exhibit a broad phenotypic spectrum ranging from completely asymptomatic individuals with normal fundus appearance to patients with clinically manifest retinitis pigmentosa [3].

Characteristic retinal findings in carriers include a tapetal-like reflex, scattered peripheral pigmentary changes (present in our patient), or more extensive retinal degeneration in manifesting carriers. In a recent publication [4] of a relatively large cohort of female carriers of RP2 disease causing variants, 29.6% had a normal fundus, 37% a tapetal-like reflex, 18.5% scattered pigmentation and 14.8% RP changes. Regarding visual function, approximately 88.9% of individuals had mild or no visual impairment. Our patient showed scattered pigmentation, radial hyperautofluorescence, mild ERG abnormalities and mild visual impairment. As a matter to remark, the availability of multimodal imaging from three affected members of the same family illustrates the intrafamilial phenotypic variability associated with this RP2 variant. While the male brother exhibited a typical RP phenotype and the mother showed advanced retinal degeneration, the proband presented only subtle structural abnormalities. Such variability in female carriers is well recognized and is thought to reflect mosaic retinal expression resulting from X-chromosome inactivation.

The development of focal inflammatory lesions consistent with acute PIC in our patient underscores the concept that inflammatory chorioretinopathy may emerge as a secondary phenomenon in eyes with pre-existing structural vulnerability, even when this vulnerability is mild, as in our case. Cicinelli et al. [1] described a series of secondary PIC arising in various degenerative and hereditary retinal disorders. In their cohort, 63% of patients demonstrated inherited retinal diseases, leading the authors to propose that RPE/Bruch’s membrane (BrM) disruption and outer retinal instability serve as local triggers for focal inflammatory activation through loss of immune privilege and exposure of sequestered antigens. Their multimodal imaging findings—hyperreflective lesions breaching the RPE/BrM complex with posterior hypertransmission—parallel those observed in the present case, further supporting its classification as secondary PIC.

In addition to the underlying dystrophy, our patient’s high myopia likely acted as an additional permissive factor. Myopic eyes exhibit mechanical stretching, microstructural RPE/BrM fragility, and choroidal thinning, all of which have been implicated in the pathogenesis of PIC even in otherwise healthy individuals. The literature describes PIC lesions preferentially forming near areas of pre-existing RPE/BrM compromise in myopia, and similar mechanisms—mechanical stress combined with barrier breakdown—may have fostered the emergence of PIC lesions in this case. Thus, the coexistence of RP2 associated Retinopathy and high myopia created a retinal environment highly susceptible to inflammatory foci.

Beyond secondary PIC, this case aligns with a growing body of evidence demonstrating that inherited retinal diseases (IRDs) can be associated with genuine intraocular inflammation. The study by Abramowicz et al. [5] reported that posterior or panuveitis with dystrophic features may mask an underlying, previously unrecognized IRD, with genetic testing refining the diagnosis in 25% of their cohort. Their findings highlight that vitritis, vascular leakage, cystoid macular edema, and mild anterior chamber inflammation are not uncommon in IRD patients and can lead to diagnostic uncertainty when inflammatory episodes precede recognition of the dystrophy.

Similarly, the comprehensive review by Hung et al. reinforces that uveitis can occur across a broad range of IRDs [6]. Proposed mechanisms include microglial activation, autoimmune responses to degenerating photoreceptor outer segments, breakdown of the external blood–retina barrier, and gene-specific pathways, such as NF-κB overactivation or defects in ciliary and outer segment structure. Collectively, these mechanisms provide a biologically plausible framework for the inflammatory episodes observed in our patient.

Apart from secondary PIC, secondary multiple evanescent white dot syndrome (MEWDS) has also been reported in the context of hereditary retinal dystrophies [2]. In MEWDS, inflammation primarily involves the photoreceptors (“photoreceptoritis”), which on OCT is typically seen as attenuation or disruption of the ellipsoid zone (EZ). Similar structural abnormalities of the EZ can also be observed in inherited retinal diseases, including X-linked retinitis pigmentosa and RP2-associated retinopathy. This issue is particularly important when examining female carriers, who often retain a relatively preserved EZ, as subtle focal alterations may represent superimposed inflammatory changes rather than primary degenerative damage. Clinically, MEWDS is usually symptomatic, often presenting with an acute scotoma, and the EZ alterations tend to be focal. In contrast, EZ loss due to genetic retinal degeneration is typically more diffuse and progressive.

Importantly, secondary PIC inflammatory flares in the context of IRD are not benign epiphenomena. As noted by Cicinelli [1] and others, secondary PIC may accelerate progression of the underlying degenerative process, promote subretinal fibrosis, or increase the risk of macular atrophy and choroidal neovascularization. Early recognition of secondary PIC is therefore essential to guide timely immunomodulatory therapy, mitigate cumulative retinal damage, and optimize long-term visual outcomes.

In summary, this case demonstrates that secondary PIC can occur in patients with RP2-associated retinopathy, particularly when compounded by high myopia, and emphasizes the need to consider inherited retinal disease in the differential diagnosis of secondary PIC. Awareness of this overlap is crucial for appropriate diagnostic evaluation, prognostic assessment, and therapeutic decision-making.

## Data Availability

No datasets were generated or analysed during the current study.

## Declarations

### AI use declaration

Generative artificial intelligence tools, including ChatGPT (OpenAI, version 4), were used during the preparation of this manuscript solely for language editing and grammatical refinement. The scientific content, interpretation of data, and conclusions are entirely those of the authors.
